# Intraspecific breakdown of self-incompatibility in *Physalis acutifolia* (Solanaceae)

**DOI:** 10.1093/aobpla/plab080

**Published:** 2021-12-23

**Authors:** Chelsea Pretz, Stacey D Smith

**Affiliations:** Department of Ecology and Evolutionary Biology, University of Colorado, 1900 Pleasant Street, Boulder, CO 80309, USA

**Keywords:** Mating system, *Physalis*, reproductive strategy, selfing, self-incompatibility, Solanaceae

## Abstract

Variation in mating systems is prevalent throughout angiosperms, with many transitions between outcrossing and selfing above and below the species level. This study documents a new case of an intraspecific breakdown of self-incompatibility in a wild relative of tomatillo, *Physalis acutifolia*. We used controlled greenhouse crosses to identify self-incompatible (SI) and self-compatible (SC) individuals grown from seed sampled across seven sites across Arizona and New Mexico. We measured 14 flower and fruit traits to test for trait variation associated with mating system. We also quantified pollen tube growth *in vivo* and tested for the presence of the S-RNase proteins in SI and SC styles. We found that seed from six of the seven sites produced SI individuals that terminated self-pollen tubes in the style and showed detectable S-RNase expression. By contrast, seed from one Arizona site produced SC individuals with no S-RNase expression. These SC individuals displayed typical selfing-syndrome traits such as smaller corollas, reduced stigma–anther distances, and a smaller pollen–ovule ratio. We also found plasticity in self-incompatibility as most of the SI individuals became SC and lost S-RNase expression roughly after 6 months in the greenhouse. While fixed differences in mating systems are known among the SI wild species and the often SC domesticated tomatillos, our study is the first to demonstrate intraspecific variation in natural populations as well as variation in SI over an individual’s lifespan.

## Teaser

This study documents the within-species breakdown of self-incompatibility in *Physalis acutifolia*, a wild relative of tomatillos. Individuals from most populations reject their own pollen, while those from one Arizona population accept self-pollen. This selfing appears due to the loss of S-RNAse proteins in the style (the tract that leads to the ovary). The Arizona population displays many characteristics associated with selfing, such as smaller and less showy flowers. In a surprising twist, the originally self-incompatible individuals began to self-fertilize as they aged, demonstrating that the capacity to self can vary across individuals and over the lifespan of a single plant.

## Introduction

Plants vary widely in mating systems, from those that exclusively outcross to those that exclusively self-fertilize to a mix of both (Goodwillie *et al.* 2005), with trade-offs for each strategy. For example, self-fertilization can result in a high number of offspring but decrease the genetic diversity of that lineage (Stebbins 1974; Holsinger 2000; Barrett 2002; Goldberg *et al.* 2010; Busch and Delph 2012). Plants have evolved multiple mechanisms to prevent self-fertilization, including the morphological separation of reproductive organs (Brys *et al.* 2014; Sedeek *et al.* 2014; Martine *et al.* 2016) and a range of genetic systems (reviewed in Takayama and Isogai 2005). These genetic systems fall into two major categories: sporophytic self-incompatibility (SSI) and gametophytic self-incompatibility (GSI) (Ride *et al.* 1999; de Nettancourt 2001; Takayama and Isogai 2005; McCubbin and Kao 2000). In SSI plants, only compatible pollen can germinate on the dry stigmas, while in plants with GSI, pollen germinates on the wet stigma and will either be terminated within the style (if incompatible) or continue to the ovules (if compatible) (Takayama and Isogai 2005). Both systems are known from multiple plant families although GSI has been studied with the most detail in Solanaceae (Franklin-Tong and Franklin 2003) and SSI in Papaveraceae (Lane and Lawrence 1993) and Brassicaceae (Nou *et al.* 1993; Ockendon 2000).

Despite strong selection to maintain SI systems at macroevolutionary scales (Goldberg *et al.* 2010), losses of SI and transitions to selfing are common across angiosperms (Stebbins 1950, 1957, 1974; Barrett 2002; Stone 2002). For instance, the model organism *Arabidopsis thaliana* transitioned from SSI-based SI to SC (Koch *et al.* 2008) with multiple breakdowns of SI due to independent genetic mutations (Nasrallah 2017). These transitions can be favoured by natural selection, for example, when populations experience limited pollinator service (Fenster and Ritland 1994), and are often accompanied by the evolution of traits that increase the efficiency of self-pollination (Luo and Widmer 2013) and decrease investment in pollinator attraction (Sicard and Lenhard 2011). Although evolutionary losses of SI are common (Igic *et al.* 2008), additional studies are needed at the population level to better understand the molecular mechanisms responsible for the breakdown of SI (Stone 2002).

Here we focus on variation in SI in the tomato family, a clade that has been fundamental to our understanding of the underlying molecular mechanisms. For instance, early studies in *Nicotiana* demonstrated that T2/S-type ribonucleases encoded by the S-locus and expressed in styles are key players in pollen recognition and rejection (Anderson *et al.* 1986; McClure *et al.* 1989; Ramanauskas and Igić 2017). Subsequent studies have revealed additional loci such as *Cullin1*, HT, Trxh and the S-locus F-box proteins involved in pollen recognition (McClure 2004; Li and Chetelat 2010; Torres-Rodríguez *et al.* 2020). Losses of expression and losses of function at these loci have contributed to natural variation in compatibility within species (e.g. Covey *et al.* 2010) as well as species-level transitions to SC in Solanaceae (e.g. Golz *et al.* 1998; Markova *et al.* 2017). While the selective forces driving losses of SI have not been studied as often in Solanaceae, range expansions have been implicated in several cases (Broz *et al.* 2017; Levin and Miller 2021).

This study examines mating system variation in *Physalis acutifolia*, a wild annual relative of tomatillo native to south-western North America. Like other Solanaceae, *Physalis* has the GSI system (Pandey 1957), with SI species presenting high allelic diversity at the S-locus (Richman and Kohn 1999; Lu 2002). Although the phylogeny is not well resolved (Whitson and Manos 2005; Zamora Tavares *et al.* 2016; Deanna *et al.* 2019), the ancestral state for the genus is definitively SI (Igic *et al.* 2006), with multiple shifts to SC in cultivated species (Menzel 1951; Azeez and Faluyi 2018; Figueiredo *et al.* 2020). The selective factors favouring transitions to self-compatibility in *Physalis* have not yet been investigated but could include range expansions in invasive species (Liu *et al.* 2006; Ozaslan *et al.* 2017), ongoing domestication (Vargas-Ponce *et al.* 2016) or fluctuations in service from their specialized solitary bee pollinators (Sullivan 1984). Polyploidy is also common in *Physalis* (Rodríguez *et al.* 2021), and may play a role in the evolution of self-compatibility (Miller and Venable 2000 but see Mable 2004). We chose to focus on *P. acutifolia* as experiments by the first author suggested that the species might comprise both SI and SC populations, providing an opportunity to study incipient breakdown. The goals of this project were to document variation in compatibility across the northern range of *P. acutifolia* and examine the mechanisms that could be responsible for the loss of SI. Using greenhouse studies of plants grown from wild-collected seed, we measured seed set and pollen tube growth in different crossing treatments and tested for S-RNase protein expression as a potential factor in controlling pollen rejection. We also quantified variation in multiple traits associated with selfing (Ornduff 1969) and determined the ploidy level of SC individuals. Collectively, these experiments present clear evidence for intraspecific variation in self-compatibility in *P. acutifolia*, accompanied by marked differences in floral biology as well as S-RNase expression.

## Materials and Methods

### Study system

**Figure 1.**
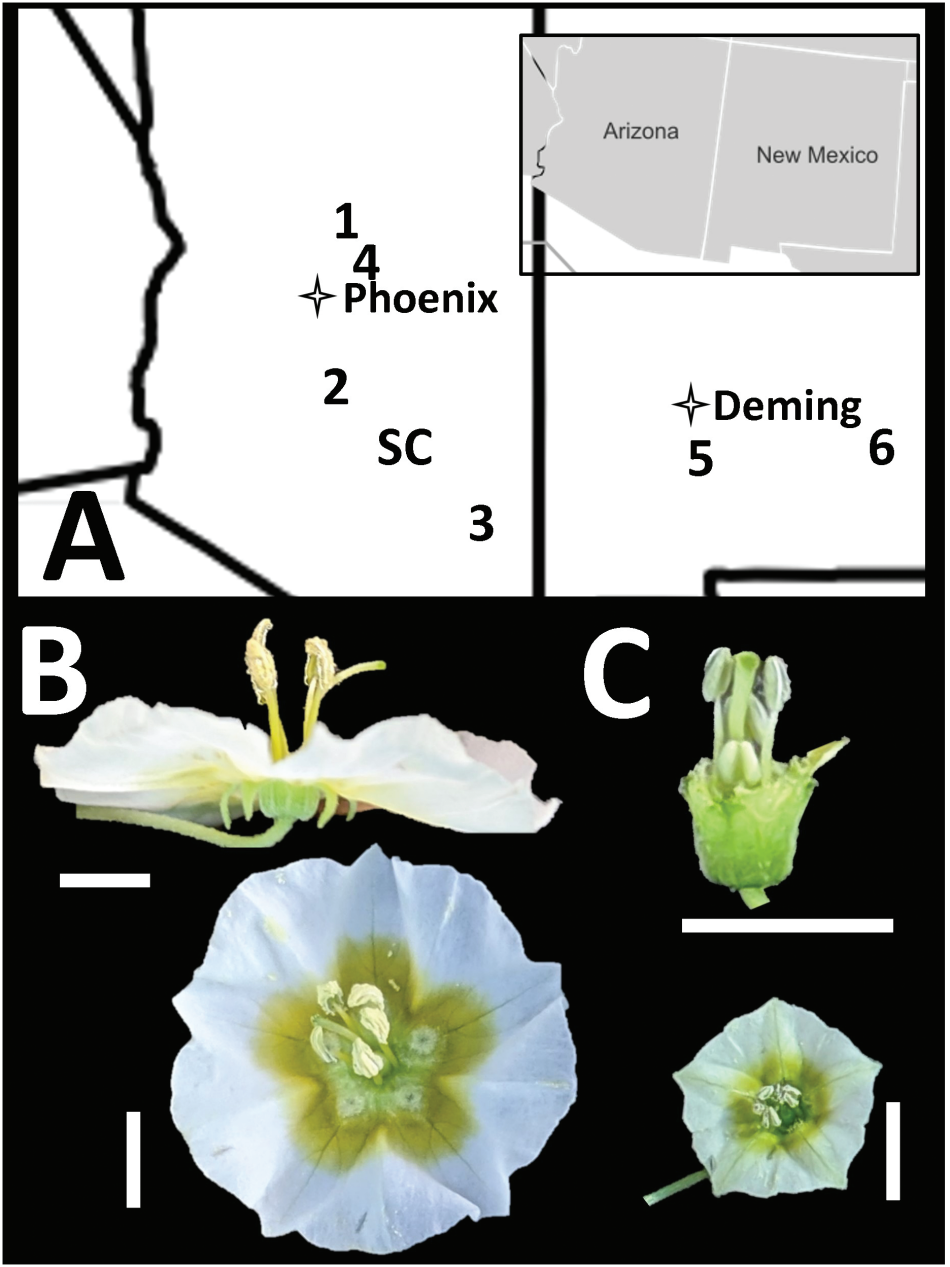
(A) Location of sampled populations in Arizona (AZ) and New Mexico (NM). Sites labeled as follows: 1 = AZ1, 2 = AZ2, 3 = AZ3, 4 = AZ4, 5 = NM1, 6 = NM2 and SC = AZ-SC (self-compatible). (B) Representative flower of sites 1–6. (C) Representative flower at AZ-SC site. In the top image, the corolla is removed to better show the carpel and stamen relationship. All scale bars are 0.5 cm.

*Physalis acutifolia* (Solanaceae) is distributed from the Southwestern United States to Northern Mexico, and with several isolated populations outside of this range in Texas, and Louisiana. It is identified by its white flowers with yellow spotting (maculae), leaves with acute apex and irregularly dentate margins, and a 10-ribbed inflated calyx enveloping the fruit. This species typically flowers from July to August, depending on rainfall (Sullivan 2004; GBIF.org 2021). For this study, seeds from a mix of individuals were collected from seven wild *P. acutifolia* populations in New Mexico and Arizona (Fig. 1). Associated voucher specimens were deposited at the University of Colorado Boulder Herbarium **[see****Supporting Information—Appendix S1****]**. Plants were grown from these seed collections in the University of Colorado Boulder greenhouses and used for the following experiments to ensure a common garden.

### Floral biology and pollination system

We made observations of pollination biology and floral phenology in the field (site NM1) and in the greenhouse to better understand the context for mating system variation in *P. acutifolia.* The flowers of *P. acutifolia* are receptive on the first day of opening, and one or two anthers will dehisce the first day (C. Pretz, pers. obs.). Over the next couple of days, the remaining anthers dehisce, and the stamens continue to elongate. After all the anthers have dehisced, the flower lasts one more day before senescing. Flowers open asynchronously throughout the day, and the corolla closes at night and reopens with sunlight.

**Figure 2.**
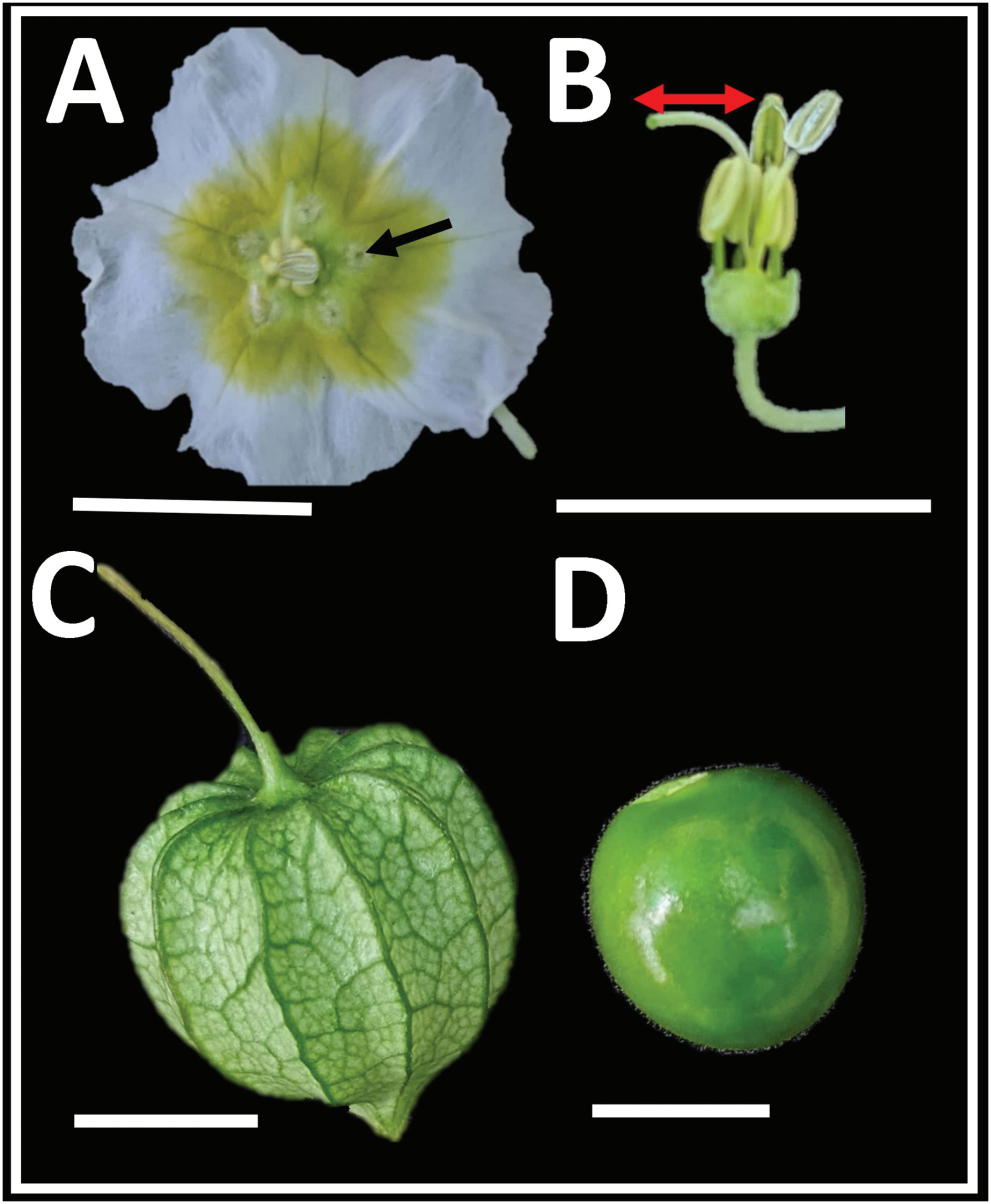
Floral and fruit morphology of *Physalis acutifolia.* (A) Flower at anthesis, with a yellow central macula and five trichome-covered nectar spots (black arrow) alternating with the stamens. (B) Reproductive organs with corolla removed. Distance between stigma and nearest dehisced anther denoted with a red arrow. (C) Mature fruiting calyx. (D) Mature fruit. Scale bars indicate 1 cm.

In the field, the first author observed that *P. acutifolia* is visited by specialized bee pollinators that collect pollen and nectar as rewards. Solitary bees (*Calliopsis* spp., Andrenidae; **see****Supporting Information—Appendix S2**) pollinate the plant in a roundabout pollination style (Endress 1997) in several sites on the northern range of *P. acutifolia* (C. Pretz, pers. obs.) These bees land on the anthers and insert their proboscis into trichome-covered nectar spots at the base of each petal (Fig. 2; **see****Supporting Information—Appendix S3**). They move in roundabout fashion from anther to anther, probing each nectar spot in turn **[see****Supporting Information—Appendix S4****]** and in so doing, touch their abdomens to the stigma as the style extends beyond the ring of anthers (Fig. 2). Studies in other *Physalis* have shown that nectar is produced at the base of the ovary (Sullivan 1984) and moves via grooves (ducts) into these nectar spots using capillary action (Vogel 1997; Dyki *et al.* 1998; Cocucci 1999; Dong *et al.* 2013).

### Greenhouse crosses

We conducted greenhouse crosses to assess self-compatibility across sampled sites. We grew 2–25 plants from seed from each of the seven sites (Fig. 1). We carried out three treatments: ‘selfed’, ‘outcrossed’ and ‘tagged’. The ‘selfed’ treatment involved the removal of undehisced anthers followed by hand-pollination with self-pollen. The ‘outcrossed’ treatment also used emasculated flowers, but involved cross-pollination from another *P. acutifolia* individual from either the same or different site. Finally, we set up a ‘tagged’ treatment, tagging flowers to track and determine if self-fertilization was occurring. Depending on the availability of flowers, we conducted 3–69 crosses per treatment per site **[see****Supporting Information—Appendix S5****]**. Roughly a month after treatment, the fruits were collected, and the seeds were counted. To avoid pseudoreplication, an average of the seeds set was taken for each individual from each treatment, and an ANOVA was conducted to determine statistical differences.

### Morphological differences between SI and SC plants

Based on the results from the crosses, which revealed both SI and SC individuals, we measured 11 traits related to pollinator attraction (corolla area, maculae area and nectar spot area), herkogamy (stamen length, anther width, style length) and breeding system (pollen and ovule counts) along with mature fruiting calyx length and width and fruit area (details about the measurements are outlined in **Supporting Information—Appendix S6**). For the seven floral traits, we measured 2–3 flowers each on 10 SI and 10 SC individuals. For the fruit traits, measurements were taken for fruits from 1 to 5 ‘outcrossed’ crosses for 16 SI and 12 SC individuals. These traits were measured from images of fresh flowers and fruits with ImageJ/Fiji (Schindelin *et al.* 2012). We measured the floral traits at two stages in development: after two anthers dehisced (‘2An’ Stage) and after all five anthers dehisced (‘5An’ Stage). Pollen counts were conducted by placing five mature anthers into 500 μL of ethanol, vortexing on high for 10 s, counting grains from three 5 μL aliquots and then taking the average. Ovules were counted by cutting the ovary in fourths, counting two fourths and multiplying the average of those two counts by four. We conducted pollen and ovules counts for three SC individuals (2–3 flowers per plant) and six SI individuals (1–2 flowers per plant). For each trait, a group average was calculated along with the standard deviation, and the means were compared with *t*-tests.

### Pollen tube growth *in vivo*

We visualized pollen tube growth *in vivo* to verify patterns of pollen growth and elongation consistent with GSI. In this system, we expect rejection of self-pollen to occur after germination, in the style. As above, we emasculated flowers before carrying out hand-pollinations. After 24 h, the styles were collected and fixed in 1:3 ethanol:acetic acid to measure pollen tube growth. For imaging, styles were first softened for 24 h in 5 M NaOH, stained in 0.001 % aniline blue and K_2_HPO_4_ for 24 h (similar to Covey *et al.* 2010), and finally mounted on a slide and imaged using Olympus IX81 Inverted Widefield Microscope. Images were processed and modified with ImageJ/Fiji (Schindelin *et al.* 2012) and Image Composite Editor (Microsoft). We aimed to image three styles for each treatment (self, outcrossed, tagged) in a total of nine images for both SI and SC groupings.

### S-RNase protein expression

We conducted western blots to test for the presence of S-RNase expression using the protocols of Murfett *et al.* (1994). Style tissue was collected from 13 individuals (six SC and seven SI), weighed and flash-frozen. Proteins were extracted by grinding frozen tissue in a Loading Sample Buffer (LSB; 125 mM Tris-HCl, pH 6.8, 4 % sodium dodecyl sulfate (SDS), 20 % glycerol, 0.01 % bromophenol blue and 50 mM dithiothreitol) with a 10 μL/1 mg LSB to tissue ratio. The sample was heated for 5 min at 99 °C, centrifuged and transferred to a new tube. We ran 10 μL of the ladder (ThermoFisher:Spectra Multicolor Broad Range Protein Ladder) and the samples on a 10 % Tris-Tricine gel (Tris, glycerol, 10 % SDS, acrylamide, BIS ACRYL) in anode (2 M Tris) and cathode (1 M Tris, 1 M Tricine, 1 % SDS) running buffer at 100 V for 2 h. The proteins were moved from the gel to a membrane with a wet transfer using Towbin buffer (25 mM Tris, 192 mM glycine, 20 % methanol, 0.2 % SDS) for 2.5 h at 100 V. Afterward, a control ponceau stain (in 5 % acetic acid) was conducted to ensure proteins were transferred. The membrane was then blocked in 1× PBS-T-4 % Blotting-Grade Blocker (Bio-Rad). Next, the membrane was probed with T-SRNA-C2 (Murfett *et al.* 1994) followed by three washes of PBS-T. Secondary antibody anti-rabbit IgG (DkxRb-003-DALP) was used, followed by washing in PBS-T and alkaline phosphate (AP) buffer. Lastly, colour development was done with a NBT/BCIP Tablet in 10 mL of AP buffer.

### Loss of SI in long-lived plants in the greenhouse

While *P. acutifolia* is a short-lived annual plant, it can persist longer in greenhouse conditions. We observed that after roughly 6 months, the SI plants started to produce self-fertilized fruits. To document this phenomenon, we quantified the seed set (13 individuals from four sites from the ‘tagged’ treatment, and seven individuals from NM1 site were ‘selfed’ with a total of 65 seed sets), imaged pollen tube growth and measured S-RNase protein expression from five individuals as described above.

### Verifying ploidy level of SC accession

Previous cytogenetic work (Menzel 1951) indicates that *P. acutifolia* is diploid (2n = 24). In order to determine if the SC population might be polyploid, root tips (ca. 1 cm) from germinating seeds were collected and used for chromosomal analysis. These radicles were placed in saturated *p*-dichlorobenzene for 2 h and moved to 3:1 ethanol:acetic acid for 24 h. The radicles were then washed in water and digested in pectinex for 1 h at 37 °C. The radicles were washed in water and then mounted on a microscope slide which was viewed under a light compound microscope. This is a simplified protocol from Rodríguez *et al.* (2021).

## Results

### Greenhouse crosses

**Figure 3.**
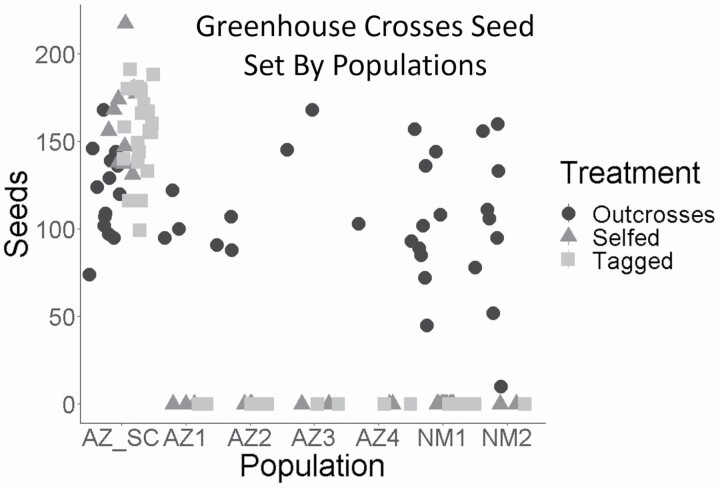
Results from greenhouse crosses of *P. acutifolia* individuals grown from seed from seven sites (Fig. 1). The average seed set per individual was plotted for each cross for each treatment type (outcrossed, tagged, selfed) from every site.

*Physalis acutifolia* individuals grown from seed from six of the seven sites were SI, while individuals from the one remaining site were SC. These results are based on 413 controlled crosses involving 50 individuals. We completed 138 crosses (30 selfed, 39 outcrossed and 69 tagged) for 25 individuals from the SC population and 275 crosses (110 self, 92 outcrosses and 73 tagged) from 25 individuals across the six SI sites (Table 1). Figure 3 shows the total set seed of each cross according to treatment and site. Only the AZ-SC site was SC. Individuals grown from seed from this site produced similar numbers of seeds in both self-fertilization (‘tagged’) and self-pollination (‘selfed’) treatments, with no significant difference in the means (*P* = 0.2679). However, there was a significant difference between the ‘tagged’ and ‘selfed’ treatments and the ‘outcrossed’ treatments with the AZ-SC individuals (*F* = 9.9659, *P* = 0.0002693). In contrast to AZ-SC, all the other sites produced seed only in the outcrossed treatment. These differences in seed set between the ‘selfed’ and ‘tagged’ treatments between AZ-SC and the SI sites are highly significant (*F* = 128, *P* < 2.2e-16 for selfed; *F* = 110.4, *P* = 2.2e-16 for tagged). While there are minor differences in average seed set in the ‘outcrossed’ treatments across sites, these were not significant (*F* = 1.32, *P* = 0.274). The SI individuals will be grouped into the SI category for the rest of the study.

**Table 1.** Greenhouse crossing results. This table shows the average seed set for individuals grown from seed from each of the seven sites. The number of individuals representing each site given in parentheses. In each treatment, the first number is the average seed set, followed by the standard error from the mean, with the number of individual crosses in parentheses.

Population	Selfed	Outcrossed	Tagged
AZ_SC (25)	150.4 ± 5.58 (30)	100.64 ± 5.37 (39)	141.26 ± 3.33 (69)
AZ1 (5)	0 ± 0 (15)	101.78 ± 3.11 (9)	0 ± 0 (9)
AZ2 (3)	0 ± 0 (15)	77.21 ± 5.81 (14)	0 ± 0 (9)
AZ3 (2)	0 ± 0 (10)	143 ± 5.71 (8)	0 ± 0 (6)
AZ4 (2)	0 ± 0 (6)	72 ± 12.83 (3)	0 ± 0 (8)
NM1 (11)	0 ± 0 (48)	92.24 ± 5.91 (38)	0 ± 0 (34)
NM2 (2)	0.12 ± 0.08(16)	104.5 ± 8.71 (20)	0 ± 0(7)
All SI localities (25)	0.02 ± 0.013 (110)	98.46 ± 2.37 (92)	0 ± 0 (73)

### Morphological differences between SI and SC individuals

Our measurements showed that SC plants have smaller flowers, smaller anther–stigma distance, smaller pollen–ovule ratio, but do not differ in the mature calyx and only slightly in fruit size compared to SI plants (Table 2). Since most of the floral differences were evident at both the 2 Anther and 5 Anther stages, we present only the 5 Anther stage in Table 2 (for the complete list, **see****Supporting Information—Appendix S6**). The only notable difference between the 2 Anther and 5 Anther stages was the distance between the stigma and nearest dehisced anther within the SI individual; while the stigma is receptive beginning at anthesis (C. Pretz, pers. obs.), the anther–stigma distance decreases from an average of 2.1 mm to only 0.9 mm because the stamens continue to elongate as the flowers mature. Overall, the largest differences between the SC and SI individuals were in the flower size, nectar spot size and the pollen–ovule ratio (Table 2). The lack of difference in fruit size was not surprising given the similarity in numbers of seeds per fruit from the crosses (Fig. 3).

**Table 2.** Differences between SC and SI plants. This table lists a subset of traits measured from self-compatible individuals (AZ_SC) and self-incompatible individuals (SI_All) with the ratio of the difference between the measurements. All floral measurements are shown at the 5 Anther stage (**see****Supporting Information—Appendix S5** for full data set). In each column, the first number is the group average, followed by the standard deviation from the mean, and lastly the sample size (number of flowers or fruits examined). *P*-value that are significant following Bonferroni correction are italicized.

	AZ_SC	SI_All	*P*-values	SI/SC ratio
Corolla size (cm^2^)	0.44 ± 0.033 (29)	2.97 ± 0.11 (38)	*1.98e-15*	6.9
Nectar spots (mm^2^)	0.03 ± 0.01 (29)	0.2 ± 0.01 (38)	*1.98e-15*	6.7
Anther width (mm)	0.52 ± 0.01 (29)	0.98 ± 0.01 (38)	*1.74e-14*	1.9
Anther length longest (cm)	0.32 ± 0.02 (29)	0.64 ± 0.01 (38)	*1.98e-15*	1.8
Stigma:anther distance (mm)	0.02 ± 0.01 (29)	0.11 ± 0.01 (38)	*1.91e-07*	4
Pollen:ovule ratio	62.84 ± 3.5 (3)	659 ± 122.27 (8)	0.0078	7.74
Calyx length (cm)	1.74 ± 0.073 (36)	1.99 ± 0.005 (63)	0.266	1.1
Calyx width (cm)	1.75 ± 0.043 (36)	1.8 ± 0.039 (63)	1	1
Fruit area (cm^2^)	1.15 ± 0.068 (36)	1.3 ± 0.036 (63)	0.000273	1.1

### Pollen tube growth *in vivo*

**Figure 4.**
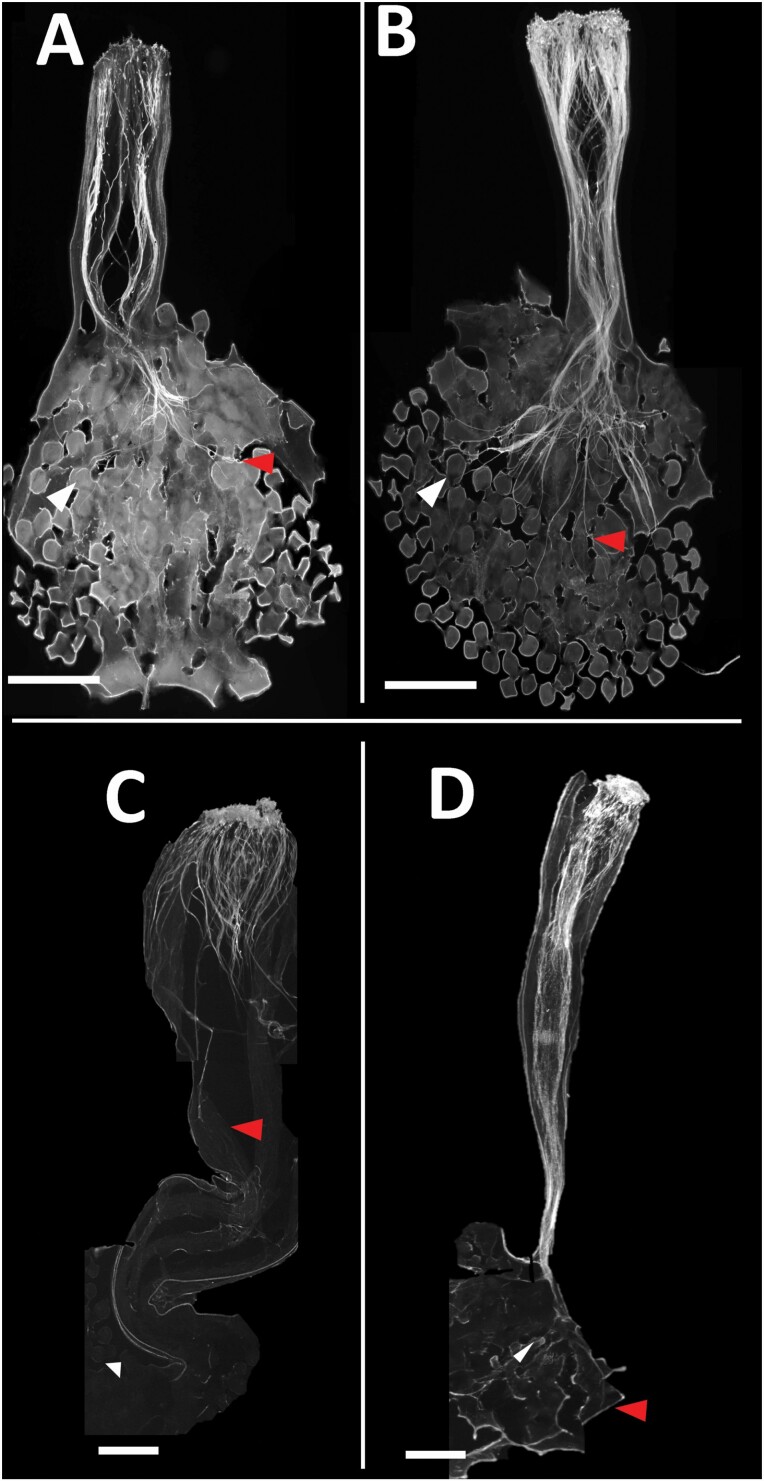
Pollen tube growth after 24 h after pollination. In each panel, the red arrow points to the end of the pollen tube growth with the white arrow pointing to an ovule. (A) Self-pollination of self-compatible AZ_SC population, showing the self-pollen reaching the ovules. (B) Cross-pollination within AZ_SC population, which is also successful. (C) Self-pollination of an individual from the NM_1 population, showing termination of pollen tube growth in the style. (D) Cross-pollination of an individual from the NM_1 population, showing the compatible pollen reaching the ovules. Scale bar indicates 1 mm.

In self-compatible plants, both self- and outcross- pollen grows to the ovary and reaches the ovules. By contrast, in SI plants, only compatible pollen from other individuals reaches the ovules while the growth of self-pollen was terminated roughly midway down the style. Representative images of this phenomenon are shown in Fig. 4.

### S-RNase protein expression

**Figure 5.**
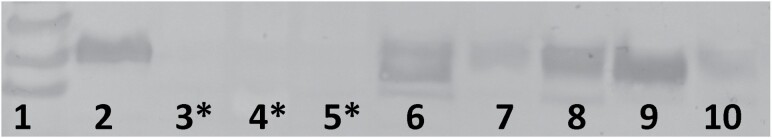
Western blot showing missing S-RNase proteins (~30 kD) in SC individuals (lanes 3–5 marked with *) (1) Ladder bottom to top ~25 kD, 35 kD, 40 kD. (2) Positive control of S-RNase antibody. (3) AZ-self-compatible individual 1 (AZ-SC-1). (4) AZ-SC-2. (5) AZ-SC-3. (6) NM1-1. (7) NM1-2. (8) AZ1-1. (9) AZ3-1. (10) AZ2-1.

Our western blot results show that SC individuals of *P. acutifolia* lack S-RNase protein expression in their styles while SI individuals show clear protein expression (Fig. 5). **Supporting Information—Appendix S7** shows that one individual from an SI site (AZ3 individual 2) lacks S-RNase expression; however, this sample was from an older individual (see next section).

### Long-lived plants in greenhouse conditions

**Figure 6.**
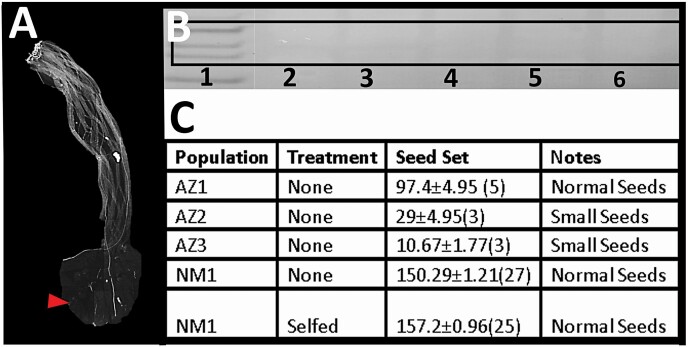
Transition from SI to SC in older plants. (A) A representative style showing self-pollen tube growth to the ovary. (B) A western blot showing the lack of S-RNase proteins in former SI individuals. (1) Ladder ~25 kD, 35 kD, 40 kD in black box, (2) NM1-3, (3) NM1-4, (4) NM1-2, (5) AZ3-1, (6) AZ2-1. (C) Results of seed set after individuals self-fertilized in the greenhouse with notes of the appearance of seeds produced.

After 6 months in the greenhouse, we observed that SI plants began self-fertilizing. We conducted additional experiments to confirm that the style tissue had stopped expressing S-RNase proteins (Fig. 6B) allowing pollen tubes to grow down to the ovary (Fig. 6A). Seed sets of these now selfing plants were examined with some individuals creating healthy seeds and other developing small fruits with small, likely unviable, seeds (Fig. 6C).

### Verifying polypoid level of SC accession

Our root squashes showed that somatic cells of *P. acutifolia* possess 24 chromosomes. Thus, the SC accession, like previously measured accessions (Menzel 1951), is a diploid **[see****Supporting Information—Appendix S8****]**.

## Discussion

### Morphological changes associated with the evolution of SC

Our results show dramatic differences in floral traits in the SC plants, similar to the suite of changes (the ‘selfing syndrome’) observed in many independent transitions to selfing throughout angiosperms (Ornduff 1969; Sicard and Lenhard 2011; Woźniak and Sicard 2018). These include reduced nectar production (Worley and Barrett 2000), reduced anther–stigma distance (Lendvai and Levin 2003) and a lower pollen–ovule ratio (Wyatt 1984; Lloyd 1965; Goodwillie 1999; Ritland and Ritland 1989). In *P. acutifolia*, we found over 6-fold reductions in flower size and nectar spot size (likely indicative of a smaller reward), both of which are consistent with fewer resources invested in pollinator attraction. A 4-fold reduction in anther–stigma distance in the SC plants suggests greater efficiency in self-pollination and self-fertilization. Indeed, the average seed set from hand-pollination with self-pollen and no treatment were very similar (150 vs. 141 seeds per fruit on average; Table 1), and both higher than for hand-pollination with outcross pollen (100 seeds per fruit). This pattern contrasts with that observed in *Witheringia solanacea*, a species within the same tribe as *Physalis*, in which SC plants suffer higher fruit abortion in self-crosses, presumably due to inbreeding depression (Stone *et al.* 2014). We did see abnormally small seeds in the SI individuals that converted to SC with age, consistent with the possibility of inbreeding depression in those SI populations.

The ecological factors driving the evolution of self-compatibility and the associated morphological changes also remain an open question in *P. acutifolia*. The documented SC site occurs in a man-made wetland area **[see****Supporting Information—Appendix S1****]**, which may allow it to persist and flower outside of the normal flowering season and thus beyond the typical foraging season of its specialized pollinators **[see****Supporting Information—Appendix S1****]**. For species or populations that do not rely on pollinators, there is no longer a reason to invest in creating large flowers and pollinator rewards (Guerrant 1989; Snell and Aarssen 2005; Eckert *et al.* 2006; Kariyat *et al.* 2021). Self-compatibility also appears to be favoured at the edges of species ranges, where there may be fewer conspecifics with whom to mate (Broz *et al.* 2017; Grossenbacher *et al.* 2017; Koski *et al.* 2019). While this SC Arizona site is well within the species range, there are several isolated populations beyond the native range that share similar morphological characteristics (e.g. small flowers) that may also be SC (C. Pretz, pers. obs.). Future work surveying mating systems and pollinator availability across the entire distribution of *P. acutifolia* could provide a clearer picture of the role of pollinator limitation and other ecological factors in driving the evolution of self-compatibility.

### Mechanisms for the loss of SI in *P. acutifolia*

Even though we cannot exclude the potential contributions of pollen factors or other genetic mechanisms, our results are consistent with the loss of S-RNase expression leading to the loss of SI in *P. acutifolia*. Our experiments showed that the SC individuals do not express S-RNases in their styles and that self-pollen tubes are not terminated and can grow to fertilize the ovules in these plants. By comparison, SI individuals express S-RNase proteins in the style and pollen tube growth is arrested in the transmitting tract, presumably as the S-RNases degrade rRNA of incompatible pollen (Franklin-Tong and Franklin 2003). While the disruption of the self-incompatibility system in the SC individuals could be due to polyploidy, we determined that is not the case in *P. acutifolia***[see****Supporting Information—Appendix S8****]**. Further support for the role of S-RNases in mediating compatibility in *P. acutifolia* comes from our finding that greenhouse-grown SI plants that transitioned to SC with old age showed a corresponding loss of S-RNase expression.

Changes in self-incompatibility with a lifespan have been documented in other species. SI has been shown to weaken with floral age both in the greenhouse (Richardson *et al.* 1990; Liao *et al.* 2015) and nature (Goodwillie *et al.* 2004). In *Solanum carolinense*, plants that set no fruit within the first 20 flowers are more likely to accept self-pollen thereafter (Travers *et al.* 2004). At a mechanistic level, this transition may be governed by S-RNase production dropping below a threshold level needed for self-pollen rejection (Qin *et al.* 2006). It is clear from our findings and these previous studies that self-incompatibility exists as a spectrum as opposed to a binary trait (Goodwillie *et al.* 2005) and that variation along this spectrum can occur not only across individuals but also over the lifespan of a plant or even a flower (Stone *et al.* 2006).

### Implications of SC transitions

The loss of SI in *P. acutifolia* may create opportunities for gene exchange with species of *Physalis*. In general, SC species are more likely to accept pollen from related SI species than vice versa, a pattern referred to as the SI × SC rule (Lewis and Crowe 1958; Hogenboom 1973; Baek *et al.* 2015). This trend extends to the population level, where SC populations may be more receptive to interspecific pollen than SI populations (Broz *et al.* 2017). Still, morphological features associated with selfing, such as shorter anther–stigma distance, may counter this effect and limit interspecific hybridization (Brys *et al.* 2016). While there have not been any documented hybrids (morphological intermediates) involving *P. acutifolia*, its range overlaps with 10 other *Physalis* species (Sullivan in prep), such as *P. hederifolia* (C. Pretz, pers. obs.). In this context, it is notable that phylogenetic analyses of *Physalis* are rife with gene tree conflict, a result that could be driven by interspecific hybridization (Whitson and Manos 2005; Zamora-Tavares *et al.* 2016; Deanna *et al.* 2019). Understanding the potential for gene flow will be critical to designing phylogenetic analyses to untangle the complex evolutionary relationships in the genus.

## Conclusion

This is the first study to examine the intraspecific breakdown of self-incompatibility in the genus *Physalis*. The SC individuals of *P. acutifolia* possess classic selfing-syndrome characteristics, such as a smaller corolla, a lack of distance between anther and stigma and a smaller pollen–ovule ratio. We also document a plausible mechanism for the transition to SC, namely the loss of stylar S-RNase protein expression. This study also revealed that individuals that persist long enough will become SC, at least in greenhouse conditions. This is important for future agronomy because SC individuals are important in breeding programs (Muñoz-Sanz *et al.* 2020). However, as in other systems (Stone 2006), multiple genes along with expression levels of S-RNases likely contribute to the breakdown of the SI system in *P. acutifolia*. Investigating the genetic architecture of SI and selfing-syndrome traits is an important next step for tracing the evolutionary steps from outcrossing to selfing in *Physalis*.

## Supporting Information

The following additional information is available in the online version of this article—

**Appendix S1.** Voucher information. All specimens were deposited at COLO. *Physalis acutifolia*. Pinal, Arizona *Pretz & Root 72* (COLO) AZ1; Pinal, Arizona *Pretz 75* (COLO) AZ2; Maricopa, Arizona *Pretz 74* (COLO) AZ3; Cochise, Arizona *Pretz 77* (COLO) AZ4; Deming, New Mexico *Pretz & Jercinovic 29* (COLO), NM1; Deming, New Mexico *Pretz & Bailey 27* (COLO) NM2; Pinal, Arizona *Pretz & Root 70* (COLO) AZ-SC.

**Appendix S2.** Pollinators of *Physalis acutifolia*. Black solitary bees (*Calliopsis* spp.) were the main pollinators observed for *P. acutifolia* at NM1. (A) A representative of a *Calliopsis* that pollinates *P. acutifolia*. (B) Pollen on *Calliopsis* leg. (C) Pollen on *Calliopsis* abdomen. Blue arrows are pointing to pollen grains in panels (B) and (C).

**Appendix S3.** Nectar spots on *P. acutifolia*. (A) Corolla with anther and carpel removed showing maculae (black arrow) and nectar spots (red arrow). (B) Close-up showing trichome-covered nectar spots, which are separated from the ovary. (C) Cross-section of a nectar spot as it would naturally hang, facing down. (D) Cross-section of the nectar spot shows that there is a pocket where nectar can accumulate. Image by S. D. Smith. (E and F) Toluidine blue-stained flower which stains polysaccharides used to determine if nectar is present. (E) The red arrows pointing to the ducts that are at the base of the ovary. (F) Ducts travel between the stamens to and nectar spots (see anatomical drawings of other *Physalis* in Vogel 1997).

**Appendix S4.** Video of *Calliopsis* pollinating a *P. acutifolia* flower.

**Appendix S5.** Greenhouse crosses. Calculations (‘Crossing Calculations’) with raw data (‘Greenhouse Crossing Data’ and ‘AgedPlant’).

**Appendix S6.** Floral and fruit measurements. Description of traits measured along with fruit and flower calculations (‘Floral & Fruit Calculations’) and measurements of all samples in ‘Floral Measurements’ and ‘Fruit Measurements’.

**Appendix S7.** Results from a second western blot (1) Ladder, (2) Positive control, (3) Negative control of LSB, (4) AZ-SC Individual 4, (5) AZ-SC Individual 5, (6) AZ-SC Individual 6, (7) NM1 Individual 3, (8) AZ3 Individual 2, (9) AZ2 Individual 2, (10) AZ4 Individual 1. C: (1) Ladder, (2) Positive control, (3) NM1-1, (4) NM1-2, (5) NM1-3, (6) AZ3, (7) AZ2.

**Appendix S8.** Chromosome squashes from the SC accession showing that SC individuals are diploid. (A) Multiple cells with chromosomes. (B) Individual cell with 24 chromosomes. Imaged by R. Deanna.

plab080_suppl_Supplementary_Appendix_S1

plab080_suppl_Supplementary_Appendix_S2

plab080_suppl_Supplementary_Appendix_S3

plab080_suppl_Supplementary_Appendix_S4

plab080_suppl_Supplementary_Appendix_S5

plab080_suppl_Supplementary_Appendix_S6

plab080_suppl_Supplementary_Appendix_S7

plab080_suppl_Supplementary_Appendix_S8

## Data Availability

All raw data are available as **Supporting Information**.

## Notes

**Populations & Communities. Chief Editor**: Jean Burns
