# Supplementary figures and images for: Intraspecific breakdown of self-incompatibility in Physalis acutifolia (Solanaceae)
Source: AoB Plants. 2021 Dec 23;14(1):plab080. doi: 10.1093/aobpla/plab080 (PMC8783618; doi:10.1093/aobpla/plab080)

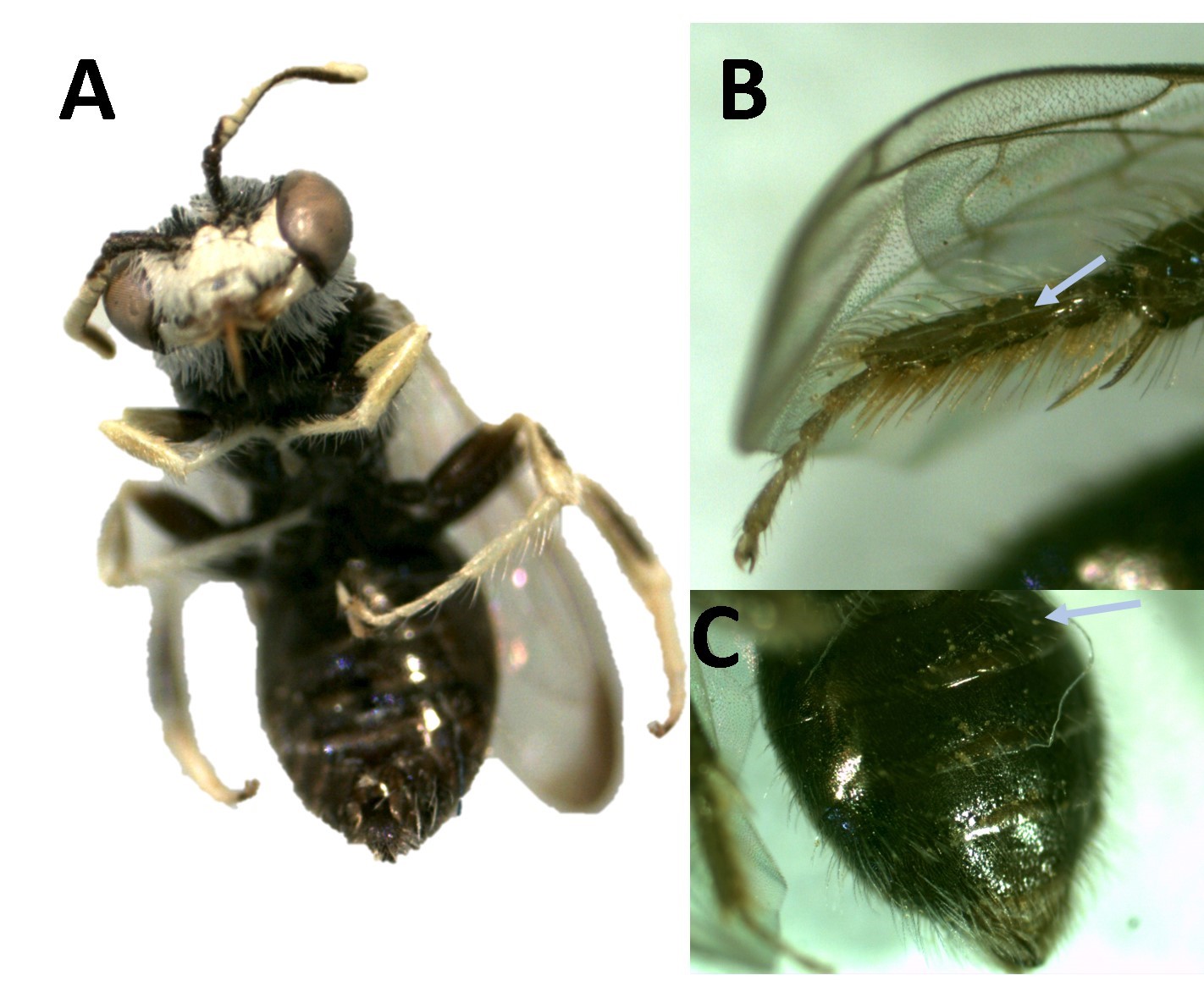

Supplement: plab080_suppl_Supplementary_Appendix_S2 [file plab080_suppl_supplementary_appendix_s2.jpeg]

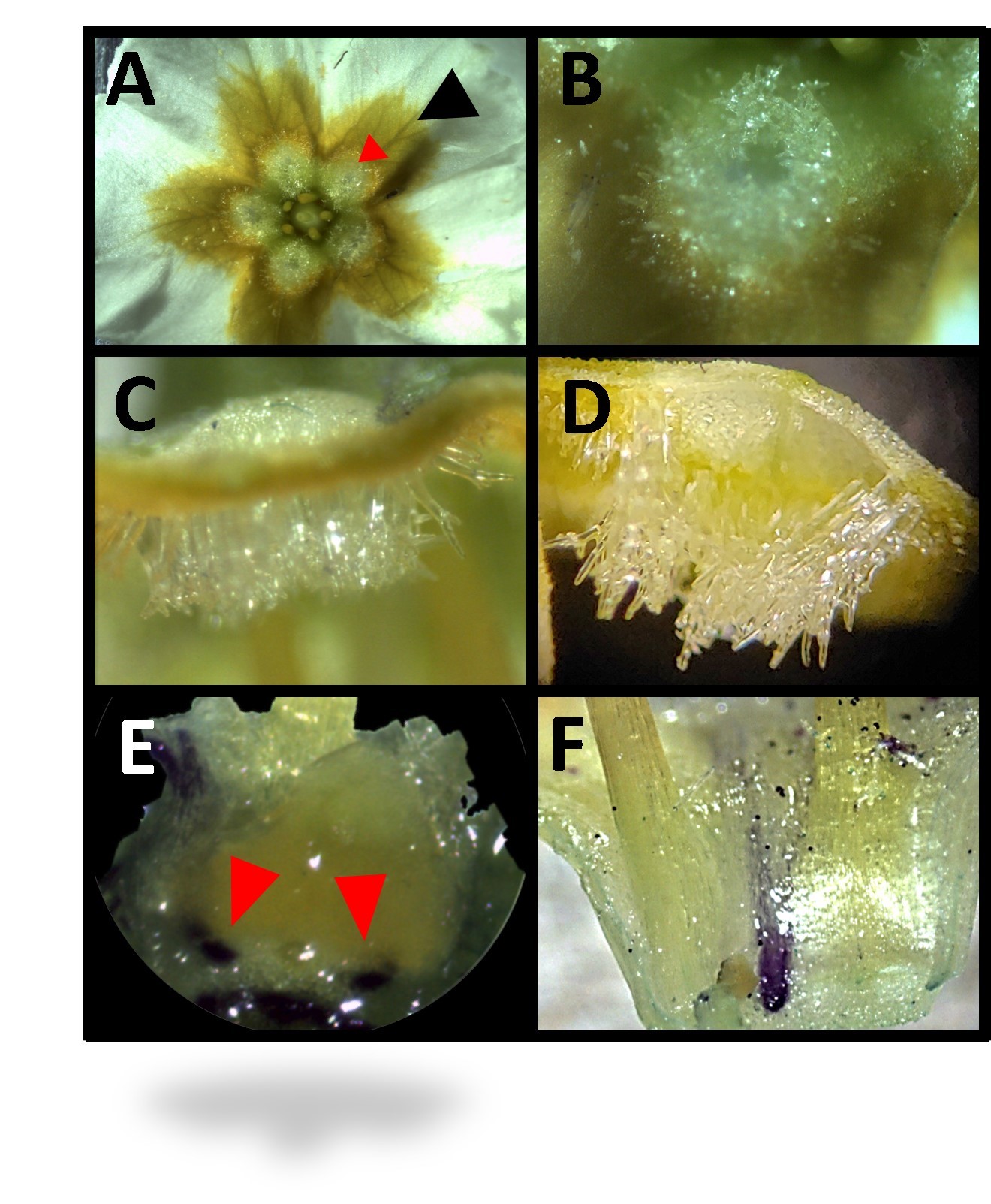

Supplement: plab080_suppl_Supplementary_Appendix_S3 [file plab080_suppl_supplementary_appendix_s3.jpeg]

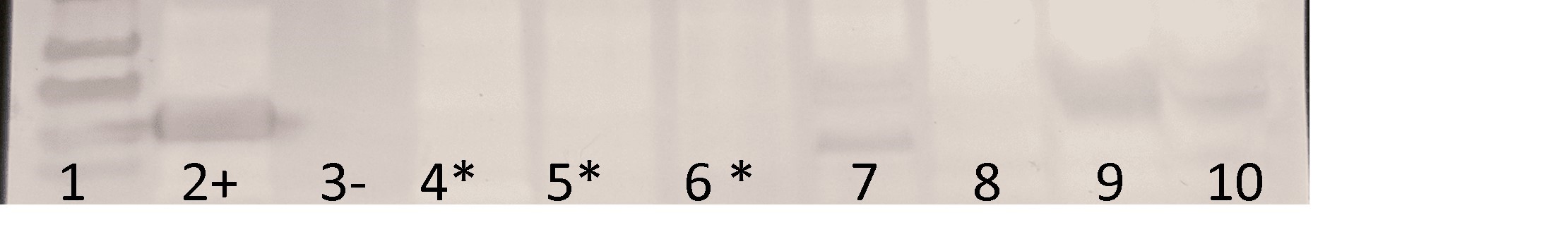

Supplement: plab080_suppl_Supplementary_Appendix_S7 [file plab080_suppl_supplementary_appendix_s7.jpeg]

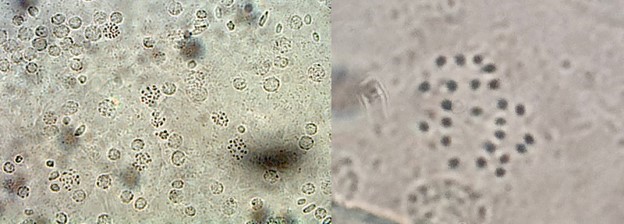

Supplement: plab080_suppl_Supplementary_Appendix_S8 [file plab080_suppl_supplementary_appendix_s8.jpeg]
